# Schlafens: Emerging Proteins in Cancer Cell Biology

**DOI:** 10.3390/cells10092238

**Published:** 2021-08-29

**Authors:** Sarmad Al-Marsoummi, Emilie E. Vomhof-DeKrey, Marc D. Basson

**Affiliations:** 1 Department of Biomedical Sciences, School of Medicine and the Health Sciences, University of North Dakota, Grand Forks, ND 58202, USA; sarmad.al.marsoummi@ndus.edu (S.A.-M.); emilie.dekrey@und.edu (E.E.V.-D.); 2 Department of Surgery, School of Medicine and the Health Sciences, University of North Dakota, Grand Forks, ND 58202, USA; 3 Department of Pathology, School of Medicine and the Health Sciences, University of North Dakota, Grand Forks, ND 58202, USA

**Keywords:** Schlafen, cancer, signaling, differentiation, invasion, proliferation, immune response

## Abstract

Schlafens (SLFN) are a family of genes widely expressed in mammals, including humans and rodents. These intriguing proteins play different roles in regulating cell proliferation, cell differentiation, immune cell growth and maturation, and inhibiting viral replication. The emerging evidence is implicating Schlafens in cancer biology and chemosensitivity. Although Schlafens share common domains and a high degree of homology, different Schlafens act differently. In particular, they show specific and occasionally opposing effects in some cancer types. This review will briefly summarize the history, structure, and non-malignant biological functions of Schlafens. The roles of human and mouse Schlafens in different cancer types will then be outlined. Finally, we will discuss the implication of Schlafens in the anti-tumor effect of interferons and the use of Schlafens as predictors of chemosensitivity.

## 1. Introduction

The Schlafens (SLFN) are a novel and poorly understood family of proteins that have chiefly been investigated for their potential roles in non-malignant cell differentiation, cell proliferation, and the immune response. However, these proteins are now increasingly believed to be important in cancer. This review will briefly summarize basic Schlafen protein biology and then outline current knowledge about the role of Schlafen proteins in cancer.

Schlafens were first discovered in mice in 1998 by Schwarz et al. [1], describing the murine proteins Slfn1, Slfn2, Slfn3, and Slfn4. These proteins have subsequently been shown to be expressed in a wide range of vertebrates, including humans [2]. Mouse Slfn1 was the first discovered Schlafen and was reported to induce thymocyte cell cycle arrest (put the cell to sleep), thereby giving rise to the name of this class of proteins from the German word “Schlafen”, which means “sleep” [1]. Later, in 2000, Slfn2 was connected to Dickkopf-1 protein (DKK1) lethality in mice [3]. In 2004, Geserick et al. [4] identified and characterized another subgroup of Schlafens in mice characterized by a C-terminal sequence motif homologous to the superfamily I of DNA/RNA helicases and were identified as Slfn5, Slfn8, Slfn9, Slfn10, and Slfn14. In 2009, genomic and phylogenetic studies conducted by Bustos et al. [5] showed that Schlafens are widely expressed in mammals and identified the Schlafen genes in humans and other mammals.

**Figure 1:**
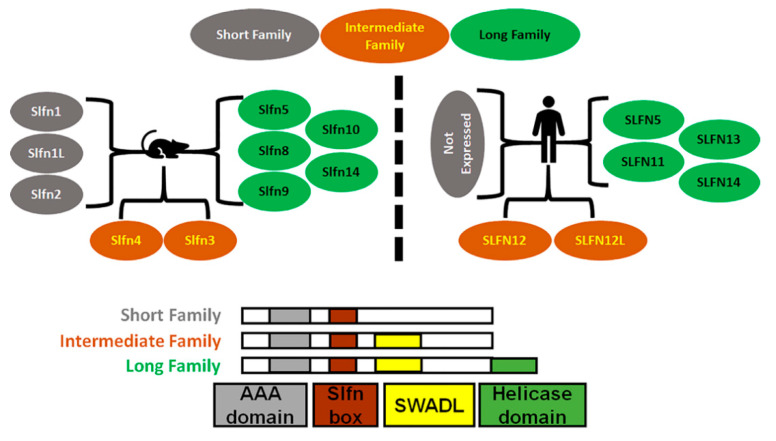
Diagrammatic representation of Schlafens family in mice and humans. Schlafens are classified into three families: short (grey), intermediate (orange), and long (green). Humans do not express short family Schlafens. All Schlafens share the SLFN box and putative AAA domains, while the SWADL domain is found in only intermediate and long Schlafens. Long Schlafens have an extra C-terminal helicase domain that harbors a nuclear targeting sequence.

Mice express ten Schlafens. These are *Slfn1, Slfn1L, Slfn2, Slfn3, Slfn4, Slfn5, Slfn8, Slfn9, Slfn10* pseudogene, and *Slfn14*. In comparison, humans express six Schlafens. These are *SLFN5, SLFN11, SLFN12, SLFN12L, SLFN13,* and *SLFN14* [2,6]. Schlafen genes are located on chromosome 17 in humans, while in mice they localize to chromosome 11 [2,6] (Figure 1).

*SLFN5/Slfn5* and *SLFN14/Slfn14* are the only direct orthologs between humans and mice. However, *Slfn3* and *Slfn4* share significant homology with *SLFN12* and *SLFN12L* and have therefore also been identified as their orthologs [7]. Phylogenic analysis suggests *Slfn8, Slfn9,* and *Slfn10* are orthologs to *SLFN13*, but there is no evidence or functional study that confirms a mouse ortholog of *SLFN11*.

Schlafens are expressed in diverse mammals, as well as in frogs and elephant fish. However, most research has focused on the role and function of Schlafens in mice, humans, and some viruses that express a viral ortholog of Schlafen identified as v-slfn. Schlafens were initially demonstrated to be differentially expressed in lymphoid tissue and thus believed to play a role in the maturation and activation of thymocytes [1]. However, further exploration has extended our understanding of Schlafen function to include roles in cell proliferation [8,9], cell differentiation [10,11], viral replication [12,13], cancer biology [14,15,16,17], and sensitizing cancer cells to chemotherapy [18,19,20,21,22,23].

## 2. Schlafen Structure

Schlafens have a unique molecular structure with no similarities to other known proteins. Sequence homology analysis for Schlafens using the NCBI database matched only Schlafens or unidentified proteins [24]. Schlafens differ in length (ranging from 337 to 910 amino acids) and structure. They are categorized into three subgroups or families based on their molecular structure and size (Figure 1). Short Schlafens have a molecular weight between 37–42 kDa, intermediate Schlafens have a molecular weight between 58–68 kDa, and long Schlafens exhibit molecular weights of 100–104 kDa, including an extra C-terminal domain [4]. All Schlafens have a Slfn-box domain, a sequence unique to Schlafens and not found in other known proteins [2]. Schlafens also contain a divergent AAA domain [2,4] and another highly conserved domain that is specific to Schlafens, known as the “SWADL” domain (the amino acid sequence Ser-Trp-Ala-Asp-Leu). This SWADL domain of unknown function is found only in short and intermediate Schlafens [25]. Long Schlafens have an extra C-terminal domain that is homologous to the DNA/RNA helicases I family [4]. In addition, long Schlafens have a nuclear localization signal and localize to the nucleus, which suggests that they may act primarily within the nucleus [25,26]. However, SLFN13, a long Schlafen, lacks the nuclear localization signal and is localized to the cytoplasm [27]. Short and intermediate Schlafens lack such signal [25,28] and have been reported to localize to the cytoplasm [25,29]. Indeed, Slfn3 retains function even when a nuclear exclusion sequence is added that specifically prevents its accumulation in the nucleus by accelerating transport back into the cytosol [28].

## 3. Role of Schlafens in Non-Malignant Biology

The Schlafen proteins biologic roles (outside of malignancy) have been chiefly investigated in mice and humans, with some attention to the viral Schlafen. Despite orthologous pairings of various Schlafens, these are all different proteins. Therefore, we will briefly summarize what is known about Schlafen biology in mice, then discuss Schlafen biology in humans, and, finally, mention viral biology.

Studies of Schlafen biology in mice have explored the roles of murine Schlafens in immunological function and immune cell differentiation [1,4], viral infections [30,31], bone development [32,33], and gut epithelial biology [29,34]. Slfn1, Slfn2, and Slfn3 are upregulated during the transition of immature T cells from CD4^+^ or CD8^+^ double-positive (DP) to single-positive (SP) [1]. Conversely, Slfn1 and Slfn2 (but not Slfn3) are downregulated in T cells upon activation and affect the growth and maturation of the T cell [1]. Both Slfn1 and Slfn2 are upregulated upon treatment with interferon-alpha (IFN-α) in melanocytes [35], lipopolysaccharide (LPS) in microglia [36], and bleomycin treatment of alveolar MLE-12 cells [5], and in splenocytes after *Listeria monocytogenes* infection [4]. Slfn1 induces cell cycle arrest by inhibiting the induction of cyclin-D1 [8]. In addition to its effect on T cells, Slfn1 also inhibits endothelial progenitor cell proliferation and tube formation [8].

Slfn2 alters the growth and differentiation of mouse hematopoietic cells [37] and is critical for T cell quiescence [1]. Indeed, Elektra mice that have loss-of-function Slfn2 point mutation are immunodeficient with decreased numbers of CD4^+^ and CD8^+^ T cells and increased apoptotic T cells [38]. In addition, Elektra mice display increased de novo synthesis of cholesterol due to increased HMG-CoA reductase activity in T cells, causing lipid accumulation inside T cells that induces chronic endoplasmic reticulum stress [39,40]. Slfn2 upregulates in macrophages upon Toll-like receptor stimulation of Activator protein-1 (AP-1) and nuclear factor kappa-light-chain-enhancer of activated B cells (NFκB) [41]. Moreover, Slfn2 is a key positive regulator of osteoclastogenesis in mice [32,33].

Slfn3 is expressed preferentially in CD4^+^ CD25^+^ Treg cells and downregulates during T cell activation by TGF-β [42]. Outside the immune system, Slfn3 also regulates enterocytic differentiation [28,43,44] and colonic mucosal growth during aging [45]. Slfn3 knockout mice show gender-specific differences in weight gain, intestinal epithelial differentiation, and impaired enterocyte differentiation after bowel resection [46]. Although Slfn3 knockout mice have been reported to have no immune phenotype [38], intestinal epithelium from Slfn3 knockout mice exhibits downregulation of the transcriptome related to immune system pathways of intestinal immune network for IgA production, leukocyte transendothelial migration, and B cell receptor signaling [46].

Slfn4 expression decreases in T cells during growth and development and increases upon stimulation of the mature T cell [1]. In contrast, Slfn4 is downregulated during macrophage differentiation but upregulated during macrophage activation [47]. Mouse Slfn4 upregulates upon treatment with IFN-α in melanocytes [35], LPS in microglia [36], and bleomycin in alveolar MLE-12 cells [5], and also inhibits myelopoiesis upon *Helicobacter* infection through Gli-dependent pathway [48,49].

Slfn5, Slfn8, Slfn9, and Slfn10 are all upregulated in melanoma cells upon treatment with IFN-α [35] and in splenocytes after infection with *Listeria monocytogenes* [4]. Slfn8 regulates the development and the proliferation of peripheral T cells [4]. Transgenic mice expressing Slfn8 within the T cell lineages have an overall decrease in thymocyte number and reduced thymus size [4]. Conversely, Slfn8 knockout mice are resistant to autoimmune encephalomyelitis [50].

In contrast to mice, humans express only intermediate and long Schlafens. SLFN5 is highly expressed in T cells and is downregulated upon T cell activation [51]. Also, SLFN5 is upregulated in astrocytes upon treatment with IFN-α [52], and in monocyte-derived dendritic cells after either IFN-α or LPS stimulation [51]. Moreover, SLFN5 acts as a transcriptional co-repressor of Signal Transducer And Activator Of Transcription 1 (STAT1) through direct interaction with STAT1 [52], and it has been identified as part of the Notch Receptor 1 (NOTCH1) nuclear interactome [53].

SLFN11 is expressed in monocytes and monocyte-derived dendritic cells [51] and is upregulated in monocyte-derived dendritic cells upon exposure to IFN-α, LPS, or rhinovirus [51]. Similar to SLFN5, SLFN11 has been identified as part of the NOTCH1 nuclear interactome [53]. SLFN11 expression in CD4^+^ cells upregulates during HIV infection in elite controllers (individuals who maintain low HIV viral load without treatment) [12], and after antiretroviral therapy of HIV patients [13], suggesting the possibility that SLFN11 may be an important factor in the suppression of HIV replication. Indeed, SLFN11 inhibits HIV replication in a codon usage-dependent manner [30]. Interestingly, equine Slfn11 inhibits equine infectious anemia virus production by a similar mechanism [54]. Moreover, SLFN11 is differentially regulated during B cell maturation, and it is epigenetically suppressed in normal germinal center B cells [55].

SLFN12 is basally expressed by monocytes, monocytes-derived dendritic cells, and T cells. Its expression decreases in monocytes in response to various differentiation stimuli [51], while it is upregulated in monocytes-derived dendritic cells by exposure to IFN-α, LPS, or rhinovirus [51]. SLFN12 is upregulated in T cells in the presence of ct-CD45 to maintain T cell quiescence [56]. SLFN12 regulates the differentiation of enterocytes by interacting with Serpin B12 and the deubiquitylases Ubiquitin Specific Peptidase 14 (USP14) and Ubiquitin C-Terminal Hydrolase L5 (UCHL5) to thereby affect the degradation of various transcription factors [29].

SLFN13 is expressed in monocytes, monocyte-derived dendritic cells, and T cells, is upregulated during the differentiation of monocyte into monocyte-derived dendritic cells [51], and further upregulated in monocyte-derived dendritic cells after exposure to IFN-α, LPS, and rhinovirus [51]. Whether higher SLFN13 expression is simply a consequence of the differentiation or actually regulates it awaits exploration.

SLFN14 expression is upregulated upon influenza virus infection in human lung adenocarcinoma cells (A549), and it inhibits viral nucleoprotein expression and translocation to the nucleus [57]. Similarly, SLFN14 inhibits the replication of varicella zoster virus (VZV) in primary human dermal fibroblasts by reducing the expression of two major VZV proteins (glycoprotein E and immediate-early protein 62) that are needed for viral replication. SLFN14 co-localizes with ribosomes to mediate rRNA endonucleolytic degradation [58] and cleave rRNA and ribosome-associated mRNA [59]. SLFN14 mutations (K218E, K219N, V220D, R223W) cause thrombocytopenia in patients [60].

In addition to mice and humans, in 2007, Gubser et al. [61] identified viral slfn (v-slfn) in camelpox virus and other orthopoxviruses. Viral slfn shares similarity with mouse short Schlafens and is one of the host-range genes responsible for host tropism in camelpox virus infection [61]. Interestingly, v-slfn recruits the lymphocytes to the lung in mice. However, these lymphocytes are less active, suggesting that viruses may utilize v-slfn to dampen the host immune response [61].

## 4. Schlafens in Cancer

**Figure 2:**
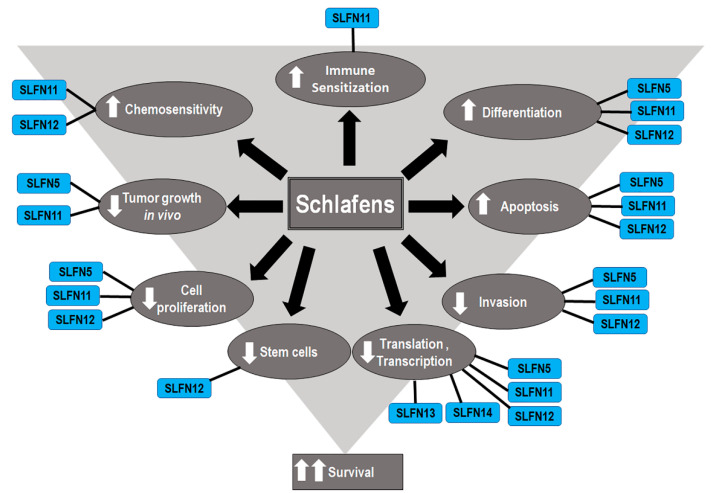
Diagram illustrates the identified effects of different Schlafens on cancer cell biology. The reduced cell proliferation, differentiation, invasion, and stem cells, in addition to the increased differentiation, immune sensitization, and chemosensitivity, all contribute to improved survival.

Although Schlafens were initially identified as proteins that influence immune cell maturation, differentiation, and responses to viral infections, recent studies have illuminated diverse potential roles for Schlafen proteins in cancer biology and malignant cell sensitivity to chemotherapy (Figure 2). The role of Schlafens in cancer has been chiefly investigated experimentally in mice and human cells and epidemiologically in humans. This review will address the effect of Schlafens in cancer in three sections. First, we will describe the role of Schlafens in the biology of different malignancies and their impact on survival, which has been chiefly investigated in humans. We will summarize the role of Schlafens in gastric cancer, malignant melanoma, lung cancer, breast cancer, gastric cancer, liver cancer, renal cancer, colorectal carcinoma, prostate cancer, central nervous system tumors, and hematological malignancies (Table 1). Second, we will summarize the role of Schlafens in the interferon immune response in cancer. Finally, we will summarize the role of Schlafens in cancer chemosensitivity to cytotoxic drugs.

## 5. Expression of Schlafens in Cancer

**Figure 3:**
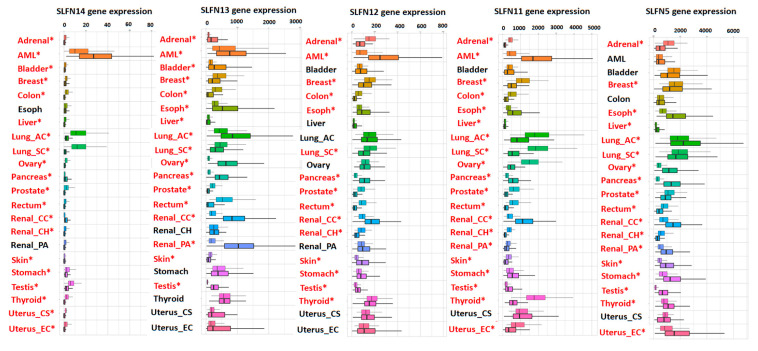
Analysis of TCGA datasets shows the expression levels of human SLFN5, SLFN11, SLFN12, SLFN13, and SLFN14 in various human tumors. Schlafens were abnormally expressed in pan cancers (right bars) by Tumor, Normal and Metastatic (TNM) plot compared to adjacent normal tissue (left bars). Significant differences by Mann–Whitney U test are labeled with red color and asterisk. Abbreviations: Lung_AC: lung adenocarcinoma; Lung_SC: lung squamous cell carcinoma; Renal_CC: renal clear cell carcinoma; Renal_CH: renal chromophobe cell carcinoma; Renal_PA: renal papillary cell carcinoma; Uterus_CS: uterine carcinosarcoma; Uterus_EC: uterine corpus endometrial carcinoma [75].

Our analysis of the TCGA pan-cancer database [75] shows cancer type-specific downregulation or upregulation of Schlafens (Figure 3). Human SLFN5, SLFN11, SLFN12, SLFN13, and SLFN14 are all downregulated in breast cancer, lung squamous carcinoma, prostate cancer, and rectal carcinoma. In contrast, these proteins are all upregulated in pancreatic and renal cell carcinoma (Figure 3). Other cancers exhibit a differential upregulation or downregulation of this family of proteins in a Schlafen-specific fashion (Figure 3).

## 6. Functions of Human Schlafens in Cancer

Human Schlafens act through different mechanisms to induce their effects in cancer cells. This section will briefly outline what is known about the mechanism of action of each.

SLFN5 has been reported to act through several different mechanisms. SLFN5 negatively regulates the expression of the matrix metalloproteinase (MMP) genes MMP-1 and MMP-13 [63]. SLFN5 modulates epithelial to mesenchymal transition by regulating the β-catenin signaling pathway [76,77]. SLFN5 also binds and represses STAT1 transcriptional activity, and subsequently inhibits interferon-stimulated gene response to STAT1 [52]. SLFN5 also inhibits AKT Serine/Threonine Kinase (AKT) and Glycogen Synthase Kinase 3 Beta (GSK-3β) phosphorylation [77] and upregulates Phosphatase And Tensin Homolog (PTEN) and AKT pathway by inhibiting the transcription of Zinc Finger E-Box Binding Homeobox 1 (ZEB1). Moreover, SLFN5 decreases cellular purine metabolites [73,74]. Finally, SLFN5 interacts with Activating Transcription Factor 4 (ATF4) and regulates the expression of Linker For Activation Of T Cells 1 (LAT1) (essential amino acids transporter), which in turn modulates the Mechanistic Target Of Rapamycin Kinase 1 (mTORC1) signaling pathway [68].

SLFN11 has been reported to increase chemosensitivity by five different mechanisms. First, SLFN11 is recruited to sites of DNA damage and interacts there with the Replication Protein A1 (RPA1) to inhibit checkpoint maintenance and homologous recombination repair [78]. Second, SLFN11 inhibits the translation of ataxia-telangiectasia mutated (ATM) and Rad3-related protein (ATR) (which are central components of the DNA damage response) by codon-specific translational inhibition [79]. Third, SLFN11 interacts with DDB1–CUL4CDT2 ubiquitin ligase to degrade Chromatin Licensing And DNA Replication Factor 1 (CDT1) and subsequently reactivates replication in response to chemotherapy leading to the collapse of replication forks and cell death [80]. Fourth, SLFN11 induces genome-wide chromatin accessibility at promoter regions during replication stress induced by DNA damaging agents [81]. Finally, SLFN11 is recruited to stressed replication forks, binds nascent DNA, and interacts with the replication helicase subunit (MCM3) without inhibiting initiation or loading of replication factor Cell Division Cycle 45 (CDC45) or the proliferating cell nuclear antigen (PCNA). This then allows SLFN11 to subsequently block replication in response to replication stress independently from ATR within 4 hours of induction of DNA damage [82].

SLFN12 binds Serpin B12 and subsequently modulates the deubiquitylases USP14 and UCHL5 to inhibit the proteasomal degradation of differentiation transcription factors such as cdx2 [29]. SLFN12 inhibits the translation of the oncogenes ZEB1 [14] and C-Myc [15]. SLFN12 is stabilized by interaction with Phosphodiesterase 3A (PDE3A) and is recruited and binds the ribosomes to exclude signal recognition peptides and subsequently inhibits translation [83]. In addition to these effects, SLFN12 has recently been identified as an RNAse that digests ribosomal RNA [84]. 

SLFN13, in contrast to other long Schlafens, is localized to the cytoplasm and acts as tRNA/rRNA-endoribonuclease that digests both tRNAs and rRNAs with a preference for tRNA, which inhibits protein synthesis [27]. In contrast, the long SLFN14 is a ribosome-associated nuclear protein that binds to the ribosomal subunits and cleaves RNA, in particular rRNA and ribosome-associated mRNA, to control mRNA turnover and protein translation [59].

## 7. Schlafens in Gastric Carcinoma

Slfn4 has been suggested as a putative biomarker for intestinal metaplasia (gastric cancer precursor) in mice [49,85]. When mice are infected with *Helicobacter felis*, SLFN4^+^ cells migrate to the stomach and express markers of the myeloid-derived suppressor cells that are responsible for the pro-inflammatory response leading to pre-neoplastic changes in *Helicobacter*-infected stomach [48]. Comparably, gastric tissues of *H. pylori*-infected patients show that SLFN12L (a human ortholog of Slfn4) co-localizes to cells that express Myeloid-derived suppressor cell (MDSC) markers [49,85]. Consequently, Slfn4 marks the myeloid-derived suppressor cell recruitment that predicts a shift of gastric mucosa to a metaplastic phenotype [49]. Slfn4 (mRNA and protein) peaks at 4 and 6 months after *H. felis* infection in the stomachs of infected mice and coincides with the onset of spasmolytic polypeptide-expressing metaplasia (SPEM) [48]. This mouse study motivated further study of human orthologs that yielded comparable results. SLFN12L immunoreactivity is not detectable in non-malignant normal human gastric mucosa without *H. pylori* infection, with no observable labeling in mucosal immune cells. However, SLFN12L^+^ immune cells are observed in *H. pylori*-infected gastric mucosa with intestinal metaplasia [49,85]. These SLFN12L^+^ immune cells are also CD15^+^ (a granulocytic-MDSC marker) and are localized near metaplastic glands. Similar to the mouse study, human SLFN12L expression increases in *H. pylori*-infected individuals with intestinal metaplasia and marks a Granulocytic-MDSC subpopulation [49,85]. Therefore, human SLFNs such as SLFN12L (orthologous to Slfn4) might serve as biomarkers for identifying pre-neoplastic transformation of the gastric mucosa in humans. These studies illustrate the potential utility of murine Schlafens in murine cancer models as a method to predict the functions of human Schlafens.

Although normal human gastric mucosa has been reported to express minimal or no SLFN5 protein [49,85], SLFN5 expression is upregulated in patients with atrophic gastritis, both intestinal and diffuse subtypes of gastric carcinoma, and in intestinal metaplasia that has progressed to gastric cancer [16]. In parallel, human normal gastric mucosa without *H. pylori* infection demonstrates negative labeling for both SLFN12L and SLFN5-expressing immune cells, but SLFN12L/SLFN5^+^ cells are observed in *H. pylori*-infected gastric mucosa with intestinal metaplasia [49,85]. Therefore, SLFN5 and SLFN12L gastric stromal expression could serve as a potential predictor of intestinal metaplasia progression to gastric carcinoma. Combining SLFN5 or SLFN12L expression levels with the histological examination of intestinal metaplasia tissue samples could substantially increase the probability of identifying patients with intestinal metaplasia that are at higher risk of progression to gastric carcinoma. The potential role of SLFN12, which shares a very high degree of similarity with SLFN12L, awaits exploration in gastric cancer.

In contrast to the apparent adverse roles of SLFN5 and SLFN12L in gastric cancer, analysis of gastric carcinomas from 169 patients suggested that high SLFN11 expression correlates with better survival, which improves when patients are treated with platinum-chemotherapy [65]. Furthermore, in vitro knockout and reactivation of SLFN11 in gastric carcinoma cell lines (MKN-1, MKN-7, MKN-45, and MKN-74) causes resistance and sensitivity to platinum-based chemotherapy, respectively [65]. Conversely, long-term oxaliplatin treatment in both gastric carcinoma cells and organoids reduces SLFN11 expression, causing oxaliplatin resistance that is reversed by reactivation of SLFN11 (with epigenetic modifying drugs). Such data suggests a role for SLFN11 downregulation in gastric cancer development and a possible clinical role for SLFN11 levels in drug selection for gastric carcinoma patients [65]. 

These results suggest opposing roles for SLFN5, SLFN12L, and SLFN11 in gastric cancer is not necessarily surprising since these are different proteins although of the same family. They also illustrate that Schlafens have a cell-type and cancer-type specific effect.

## 8. Schlafens in Malignant Melanoma

Knockdown of Slfn2 or Slfn3, but not Slfn5, increases proliferation and anchorage-independent growth in murine melanoma cells [35]. Human primary melanocytes detectably express SLFN5, SLFN11, SLFN12, SLFN13, and SLFN14, with SLFN5 being the highest expressed Schlafen [62]. Although SLFN11, SLFN12, SLFN13, and SLFN14 are expressed comparably in primary melanocytes and malignant melanoma cells, the high expression of SLFN5 in primary melanocytes is suppressed at both mRNA and protein levels in malignant melanoma cells. [62] Further SLFN5 knockdown promotes anchorage-independent growth and invasion in human malignant melanoma cells [62]. This suggests a specific role of SLFN5 in the pathogenesis of melanomas and the importance of SLFN5 downregulation in promoting melanoma tumorigenesis. More work is needed to elucidate or exclude any potential role of other Schlafens in malignant melanoma.

## 9. Schlafens in Renal Carcinoma

Higher SLFN5 expression correlates with better overall survival of patients with renal cell carcinoma [63]. In vitro overexpression of SLFN5 reduces the motility and invasiveness of malignant human renal cell carcinoma (RCC) cells by negatively regulating the expression of MMP-1 and MMP-13 [63]. Although SLFN5 expression has been studied in normal and cancerous renal cells, whether SLFN5 expression is downregulated or upregulated in renal carcinoma compared to adjacent non-tumor tissue has not been previously reported. Our analysis of the TCGA dataset (Figure 3) suggested that SLFN5 expression is significantly downregulated in RCC and renal papillary cell carcinoma, but it is upregulated in renal chromophobe cell carcinoma (Figure 3). Unlike SLFN5, SLFN11 expression is reduced in renal tumors compared to the adjacent non-tumorous tissue in both papillary renal cell carcinoma and chromophobe renal cell carcinoma [64], raising the possibility that SLFN11 downregulation may contribute to tumorigenesis in both papillary and chromophobe RCC. SLFN11 expression is also strongly correlated with the expression of CD47, a cell surface marker that sensitizes cancer cells to chemotherapy and radiotherapy, in RCC [86], while exogenous SLFN11 overexpression sensitizes CD47 negative cancer cells to radiotherapy [86].

These studies suggest a positive effect on survival for expression of both SLFN5 and SLFN11 in human RCC. However, the statistical effects of SLFN5 expression on survival in the subtypes of renal cancers (papillary renal cancer and chromophobe renal cancer) await exploration. Either SLFN5 or SLFN11 downregulation might be a part of the tumorigenesis in RCC, which awaits further study.

## 10. Schlafens in Colorectal Carcinoma

Colorectal cancer (CRC) is one of the most common cancers. Our clinical approach to this disease has included more aggressive surveillance, less invasive surgical techniques, and interference with Vascular Endothelial Growth Factor (VEGF) and Endothelial Growth Factor (EGF) receptors in metastatic disease [87,88]. A better understanding of the molecular biology of these tumors is certainly desirable. Colon cancer tissues show consistently negative immunohistochemical labeling for SLFN11 [64]. SLFN11 is methylated in more than half of the examined samples of human CRC, with no similar methylation seen in non-cancerous colorectal mucosa [22]. Hypermethylation of the SLFN11 CpG promoter inactivates SLFN11 gene expression in cancer cells [21], which might contribute to the reported negative SLFN11 labeling in colon cancer. Moreover, methylation of SLFN11 (which correlates with SLFN11 expression) significantly correlates with age, poor 5-year overall survival, and poor 5-year relapse-free survival in colon cancer [22]. Therefore, SLFN11 methylation (which inversely correlates with SLFN11 expression [21]) could serve as an independent prognostic factor for overall and relapse-free survival in colon cancer [22]. In vitro, exogenous expression of SLFN11 in CRC cell lines that express low levels of endogenous SLFN11 suppresses the aggressive behavior of such cells by reducing cell proliferation and colony formation and reducing the expression of cyclin D1 and cyclin E1. Conversely, expression of cyclin D1 and cyclin E1 increases after knockdown of SLFN11 in DKO colon cancer cells that express high endogenous SLFN11 [22]. Interestingly, SLFN11 sensitizes RKO, DLD1, and SW620 colorectal cancer cells to cisplatin and reduces in vivo tumor growth in mice [22], suggesting a favorable role for SLFN11 in CRC. As for gastric cancer, SLFN11 expression could be a prognostic marker for gastric and colon cancers. Although SLFN11 is a long SLFN that targets to the nucleus, SLFN12 is an intermediate SLFN that acts in the cytosol (Figure 1). Overexpressing SLFN12 in human colon adenocarcinoma cells induces the differentiation of such cells by modulating the activity of the deubiquitylases USP14 and UCHL5 [29].

Interestingly, the effect of Schlafens on colon cancer extends beyond the human Schlafens, as exogenous expression of mouse Slfn3 in human CRC cell lines that lack Slfn3 induces G0/G1 arrest by downregulating Cyclin D1 expression and reducing phosphorylation of retinoblastoma (pRB) protein [45]. Slfn3 expression in human colon cancer cells induces differentiation, inhibits TGF-α expression, and reduces the cancer stem cell population within a heterogeneous cell population, and inhibits colonosphere formation [11,28,43]. In addition to influencing native differentiation, Slfn3 overexpression modulates the response to chemotherapy, downregulating the expression of ATP Binding Cassette Subfamily G Member 2 (ABCG2), which transports chemotherapeutic drugs, and stimulating apoptosis in response to additional folic acid, fluorouracil, and oxaliplatin (FOLFOX) treatment in FOLFOX-resistant HCT-116 and HT-29 cells [11]. Although Slfn3 is not expressed in humans, such findings might predict the role of human orthologs of Slfn3 (SLFN12) in colon cancers and further support the need to explore the potential role of SLFN12 in colon cancer.

## 11. Schlafens in Lung Cancer

SLFN12 correlates with survival in patients with lung adenocarcinoma but not in patients with lung squamous cell carcinoma [15]. In vitro, overexpressing SLFN12 in lung adenocarcinoma cells reduces proliferation by inhibiting c-myc translation, with no similar effect on c-myc or cell proliferation in lung adenocarcinoma cells [15], demonstrating a specific SLFN12 effect in one distinct subtype of lung cancer (lung adenocarcinoma).

SLFN5 protein expression positively correlates with the overall survival in non-small cell lung cancer (NSCLC) [66]. Although both SLFN5 and SLFN11 are downregulated in NSCLC vs. adjacent non-cancerous tissue specimens [64,66], higher SLFN5 expression is detected in early-stage NSCLC tissues [66]. SLFN5 expression is highly correlated with the clinicopathological characteristics of NSCLC (TNM classification) [66], suggesting that SLFN5 may contribute to tumorigenesis and progression of lung cancer. However, studies of SLFN5 have not yet distinguished among the different histological subtypes of NSCLC, which may be worthwhile given the differential relevance of SLFN12 in NSCLC subtypes [15].

In vitro, SLFN5 knockdown in A549 adenocarcinoma human alveolar basal epithelial cells upregulates metalloproteinases, specifically matrix metalloproteinase MMP9. This facilitates cell invasion and, indeed, increases A549 cell two-dimensional migration [66], although true invasion was not addressed in that study. This would be important to address since true invasion is more complex than cell motility and requires a balance between matrix metalloproteinase activities and TIMP levels that is not necessarily predictable by the expression of a single matrix metalloproteinase. Guo et al. [76] similarly reported that SLFN5 overexpression induced epithelial to mesenchymal transition in A549 cells, promoting nuclear translocation of β-catenin and expression of Snail and vimentin, while downregulating E-cadherin and increasing both two-dimensional migration and invasion [76]. Conversely, A549 cells, in which SLFN5 has been reduced, displayed epithelial morphology with upregulated E-cadherin and downregulated vimentin and decreased cell migration and invasion [64]. In contradiction to the findings of Guo et al. [76], Wan et al. [77] reported that lung adenocarcinoma A549 cells have high SLFN5 expression. Furthermore, reducing SLFN5 in these cells activated the β-catenin signaling pathway, enhancing Metallothionein 1 (MT1)-MMP expression and increasing migration and invasion of A549 lung adenocarcinoma cells in vitro and even in vivo, when SLFN5 knockout A549 cells were injected in chorioallantoic membrane of the chick embryo [77]. Indeed, Wan and colleagues [77] reported similar findings in MCF7 (breast) and HCT-116 (colon) cancer cell lines that are also less invasive [77]. The discrepancy in the reported effect of SLFN5 knockdown in A549 lung adenocarcinoma cells between these studies could reflect differences in cell passage or type between the two laboratories with phenotypic drift. However, it is noteworthy that Guo et al. [76] performed stable SLFN5 transfection/knockdown of A549 cells with a lentiviral vector while Wan et al. [77] used transient transfection/knockdown. The adaptation of A549 cells to the continuous stable loss of SLFN5 or off-target effects of the transient knockdown could affect other Schlafens. Manipulating levels of one Schlafen protein may affect others by feedback loops that await elucidation [89]. Therefore, it would be interesting and potentially explanatory to examine the expression of other endogenous Schlafen proteins in the experiments described by Guo et al. [76] and Wan et al. [77].

SLFN11 expression is prognostically significant and correlates with improved patient outcomes after adjuvant chemotherapy in lung squamous cell carcinoma [67]. Furthermore, in lung squamous cell carcinoma, but not lung adenocarcinoma, SLFN11 expression correlates with the expression of CD47 (a cell surface molecule that sensitizes cancer cells to radiotherapy and chemotherapy) [86]. This indicates a role of SLFN11 in the radiosensitivity of lung squamous cell carcinoma. Although not examined in lung cancer cells, increasing SLFN11 expression in CD47 knockout Jurkat cells restored their radiosensitivity [86].

## 12. Schlafens in Prostate Cancer

The role of each Schlafen has not been studied extensively in all subtypes of prostate cancer. Most studies have examined the role of Schlafens in castration-resistant prostate cancer. The expression of some Schlafens seems favorable, while others correlate with more aggressive prostate cancer behavior.

SLFN5 is identified as an androgen receptor-regulated protein in castration-resistant prostate cancer (CRPC). CRPC tumors exhibit high SLFN5 expression, which correlates with poor patient outcome [68]. SLFN5 is also among thirty upregulated genes in docetaxel-resistant C4-2B and LNCaP prostate cancer cells [90]. In vitro, SLFN5 depletion strongly reduces tumor growth in CRPC by decreasing intracellular levels of essential amino acids and impairing mTORC1 signaling [68]. Therefore, as in gastric cancer, SLFN5 expression is an unfavorable prognostic predictor for CRPC. The potential role of SLFN5 in other subtypes of prostate cancer awaits exploration.

Circulating tumor cells (CTCs) from metastatic CRPC patients show SLFN11 gene methylation, suggesting the need for SLFN11 silencing in prostatic cancer cells to gain metastatic ability [91]. SLFN11 re-upregulates in the circulating tumor cells upon treatment with platinum-based drugs, implicating SLFN11 upregulation in the CRPC response to platinum therapy [69]. In contrast, SLFN11 is not overexpressed in neuroendocrine prostate cancer patients treated with platinum, and it has remained lower than CRPC with adenocarcinoma histology [69], indicating that SLFN11 upregulation in response to platinum-based chemotherapy is specific to prostatic adenocarcinoma. Unlike SLFN5, no significant correlation is observed between SLFN11 expression and overall survival, but a longer radiologic progression-free survival (rPFS) is seen in CRPC patients with adenocarcinoma histology with SLFN11 overexpression vs. low SLFN11 expression in patients that are treated with platinum-based chemotherapy, and no similar effect on rPFS is observed in neuroendocrine prostate cancer [69]. Also, SLFN11-overexpressing prostate cancer patients show less serum prostatic specific antigen (PSA) than patients with low SLFN11 tumors [69]. SLFN11 expression increases the sensitivity of prostate cancer cells to platinum-based drugs in vitro, and knockout of SLFN11 in human prostate cancer organoids increases the resistance to Cisplatin and Olaparib (Poly (ADP-ribose) polymerase inhibitors, PARPi) in prostatic organoids [69].

Similar to what has been observed in lung cancer, CD47 positively correlates with SLFN11 expression in prostate carcinoma but not in normal prostate tissue [86]. Moreover, knockdown of CD47 in prostate cancer PC3 cells reduces both SLFN11 mRNA and protein levels, indicating SLFN11 is a target of CD47 in prostate cancer cells [86]. Although SLFN11 knockdown does not protect CD47-null PC3 cells from the effect of ionizing radiation as in other cancer cells, the low SLFN11 in CD47-null PC3 cells reduces the sensitivity of these cells to DNA damaging agents such as etoposide [86]. In addition, SLFN11 protein levels increase 24 hours after treating wild type PC3 cells with sublethal doses of rocilinostat, entinostat (HDACs), or etoposide. CD47-null PC3 cells lack SLFN11 expression induction by such drugs, indicating a CD47-dependent effect of HDAC inhibition on SLFN11 expression in PC3 cells [86]. Moreover, CD47 regulates SLFN11 expression in prostate cancer through promotor methylation [86].

Unlike the nuclear-localized SLFN5 and SLFN11 [25], the intermediate family SLFN12, which lacks nuclear import signal and localizes to the cytoplasm [10], also has a differentiation effect in prostate cancer cells [92]. Overexpressing SLFN12 in prostate cancer LNCaP and PC-3 cells induces the differentiation of these cells as indicated by the reduced PSA expression and increased dipeptidyl-peptidase-4 and E-cadherin expression independently of the other known differentiation pathways [92]. Although this SLFN12 study did not conduct survival analysis in prostate cancer, better differentiation of cancer cells is known to have a better outcome; therefore, SLFN12 expression is predicted to correlate with better prostate cancer outcomes [92].

## 13. Schlafens in Liver Cancer

Only SLFN5 and SLFN11 have been investigated in liver cancer. SLFN11 mRNA and protein are downregulated in hepatocellular carcinoma (HCC) vs. non-tumor liver tissues [71]. Low SLFN11 expression correlates with shorter overall survival, higher recurrence rates, and more aggressive clinicopathologic characteristics in HCC patients [71]. SLFN11 expression is negatively correlated with high serum alpha-fetoprotein levels (a marker widely used to detect HCC), tumor size, microvascular invasion, and advanced stage [71]. L-02 normal liver cells have higher SLFN11 expression than HCC cell lines, and overexpressing SLFN11 in HCC cell lines (HCCLM3, Hep3B, SMMC-7721, and PLC/PRF/5) inhibits cell proliferation, migration/invasion, induces apoptosis, and reduces HCC growth and metastasis in vivo. All these effects are attenuated by SLFN11 knockdown [71].

SLFN5 is also downregulated in hepatocellular carcinoma. Lower SLFN5 expression correlates with poor survival and more aggressive clinicopathological features (TNM) of the disease [70]. As for SLFN11, normal liver cells (L-02) have higher SLFN5 expression (mRNA and protein) than HCC cell lines (SMMC-7721, BEL-7402, Huh 7, HepG2 cells) [91]. Mechanistically, SLFN11 physically binds RPS4X and attenuates both S6 and eIF4E phosphorylation, blocking mTOR signaling [71]. Overexpression of SLFN11 in an orthotopic mouse model reverses HCC progression and metastasis [71]. Hence, SLFN5 and SLFN11 may be used as prognostic biomarkers. Furthermore, SLFN11 may be a tumor suppressor that blocks mTOR signaling in HCC and therefore a potential therapeutic target in HCC patients.

## 14. Schlafens in Esophageal Cancer

High SLFN11 expression is correlated with a better prognosis in esophageal squamous cell carcinoma patients [72]. The prognostic value of high SLFN11 expression is most prominent in patients at clinical stages II and III who received definitive chemoradiation therapy (combination of nedaplatin, 5- fluorouracil, and irradiation) [72]. Although this has not been investigated in vitro in esophageal cancer cells, the SLFN11-positive leukemic cell lines K562 and CCRF-CEM are more sensitive to platinum derivatives, but not 5-fluorouracil compared to SLFN11-knockout cells. [72] Thus, it is possible that the observed SLFN11-dependent improvement in clinical outcome in esophageal squamous cell carcinoma may reflect the improved response of tumors expressing high SLFN11 to platinum but not 5-fluorouracil. Therefore, SLFN11 expression could be a prognostic marker for esophageal squamous cell carcinoma and a potential biomarker for therapy selection in esophageal squamous cell carcinoma [72]. No data is available regarding the role of Schlafens in esophageal adenocarcinoma, which still awaits exploration.

## 15. Schlafens in Breast Cancer

Two Schlafens have been reported to similarly correlate with breast cancer biology but by different mechanisms. Expression of both the long SLFN5, which targets to the nucleus, and the intermediate SLFN12, which targets to the cytosol, is correlated with survival, tumor growth, and metastasis in triple negative breast cancer [14,73,74]. SLFN5 expression has also been reported to correlate inversely with metastasis in Luminal A breast cancer [73]. Although overexpression of either SLFN5 or SLFN12 in invasive breast cancer cell line (MDA-MB-231) induces mesenchymal–epithelial transition (MET) and increases E-cadherin, and reduces vimentin expression by modulating ZEB1, they do so through different mechanisms. SLFN5 inhibits the transcription of ZEB1 through direct promoter binding [73]. It subsequently upregulates the transcription of PTEN (a known cancer suppressor gene), induces molecular changes in the downstream AKT pathway, and proliferation/apoptosis in both ER^+^/PR^+^ MCF-7 and ER^-^/PR^-^ MDA-MB-231 cells [74]. SLFN5 also decreases purine metabolites (inosine, xanthine, and hypoxanthine) in breast cancer cells [74]. In contrast, SLFN12 inhibits ZEB1 translation without transcriptional inhibition, induces CD44^+^/CD24^-^ stem cell differentiation, and reduces the proliferation of triple negative breast cancer cells but not the ER^+^/PR^+^ MCF7 cells [14]. Such differences in mechanistic action might be attributed to the different localization of SLFN5 and SLFN12, as SLFN5 localizes to the nucleus [25], while SLFN12 localizes to the cytoplasm [29] and lacks the nuclear localization signal that is present in SLFN5. Interestingly, deletion of the C-terminal of SLFN5 abolishes the SLFN5 effect on ZEB1, indicating that the activity of SLFN5 requires the C-terminal domain that harbors the nuclear import signal [73]. In contrast, point mutations that disrupt the central domain of SLFN12 (D233Q, Y236F) abolish SLFN12 differentiating effect in Caco-2 cells [29].

Such data encourage exploring the role of Schlafens in various subtypes of cancer rather than aggregating all types of cancer in a given organ or tissue together and suggests SLFN5 and SLFN12 as potential therapeutic targets for breast cancer.

In contrast to the reported positive effects of SLFN5 and SLFN12 in breast cancer, high SLFN11 expression correlates with more aggressive breast cancer tumors with signs of immune activation (basal-like phenotype, higher histological grade, younger age), while lower SLFN11 expression is noted in the luminal, less aggressive neoplasms characterized by low immune activation [17]. Moreover, SLFN11 positively correlates with markers of lymphocytic tumor infiltration such as CD3 and CD8 in breast cancer [17]. Interestingly, similar to SLFN5 and SLFN12, SLFN11 overexpression is independently associated with a better prognosis. However, patients with high SLFN11 and undergoing hormone therapy have a short-term worse prognosis in the first two years [17]. This could be because high SLFN11 breast cancers are mostly ER-negative, basal-like phenotype that are known for their poor response to hormonal therapy [17,93]. Another study examined the patient-derived xenografts (PDXs) response to DDA and has shown SLFN11 is significantly elevated in metastatic tumors compared to non-metastatic ones, and high SLFN11 metastatic PDXs showed better response to therapy with DNA damaging agents [94].

Unlike the observation with SLFN11, SLFN12 expression is correlated with endocrine therapy sensitivity in estrogen-positive breast cancer, as SLFN12 is one of the 60 differentially methylated region (DMR) genes in breast cancers with endocrine resistance. The CpG methylation of promotor region of SLFN12 gene is correlated with lower mRNA expression and subsequently endocrine therapy resistance [95].

## 16. Schlafens in Central Nervous System Cancers

Strikingly, all the examined human Schlafens may be poor prognostic indicators in neurological tumors. In glioblastoma multiforme (GBM), high levels of all human Schlafens (SLFN5, SLFN11, SLFN12, and SLFN13) are correlated with shorter overall survival of patients [52]. Specifically, the expression levels of SLFN5, SLFN11, and SLFN12 are positively correlated with glioma grade, highest in the more aggressive grade IV compared to grade I, II, or III tumors [52]. Knockdown of SLFN5 in GBM cells (LN18 and U87MG) reduces proliferation, anchorage-independent growth, invasiveness, and tumorigenesis in vivo when cells are injected in mice [52].

## 17. Schlafens in Hematological Malignancies

Although Schlafens were discovered initially in immune and hematological cells with varying expressions during different stages of differentiation and growth [51,56], the role of Schlafens in leukemia development and progression has not been examined extensively.

Silencing Slfn2 prevents the growth of pre-leukemic T cells in T cell acute lymphoblastic leukemia (T-ALL), which is induced by the intracellular domain of NOTCH1 (ICN1) [96] and attenuates the development and the progression of T-ALL in mice [96]. Furthermore, the loss of function of Slfn2 in Elektra mice protects these mice from ICN1-induced T-ALL [96], while knockdown of Slfn2 in EL4 (a murine T cell lymphoma cell line) reduces its proliferation, attenuates T cell lymphoma development when injected in mice, and improves survival [96]. Part of these positive effects is due to p53 pathway activation [96].

A novel missense mutation (E261Q) of SLFN12 gene has been discovered in CD4^+^ T cells of patients with Sezary syndrome (T cell cutaneous lymphoma) [97]. Overexpressing SLFN12 in the continuously proliferating Jurkat cells (T cell leukemia cell line) reduces the overall cellular growth and viability [56].

SLFN11 modulates the sensitivity of HAP1 cells (derived from chronic myelogenous human leukemia) to T cell mediated cytotoxicity [98]. Interestingly, silencing SLFN11 expression reduces the sensitivity of HAP1 to both IFN-γ and DNA damaging agents [98].

## 18. Role of Schlafens in the Antitumor Effects of Interferons

The interferons have long been known to have anti-tumor effects, particularly in hematological cancers [99,100]. Although interferons stimulate a wide array of genes responsible for the complex responses of cancer cells to interferons, the exact mechanistic pathway through which interferons act remains unclear.

Schlafens are interferon regulatory factor (IRF-1) dependent [4] interferon-stimulated genes [26]. Interferon (IFNα) induces the expression of Slfn1, Slfn2, Slfn3, Slfn4, Slfn5, and Slfn8 in B16-F1 malignant murine melanoma cells and murine renal cell carcinoma cells (RCC) [35]. Knockdown of Slfn2 or Slfn3, but not Slfn5, increases cell proliferation and anchorage-independent growth and reduces the antiproliferative effect of interferon in murine melanoma cells [35]. However, reducing Slfn5 in mouse renal cell carcinoma cells increases cell proliferation and anchorage-independent growth, and reduces the antiproliferative effect of interferon [35].

Interestingly, IFN-I substantially upregulates SLFN5 in malignant melanoma cells [62], in normal renal proximal tubule epithelial cells (RPTEC), and human renal adenocarcinoma cells (786-O and ACHN RCC cells), with minimal or no induction of other human Schlafens [62]. This suggests a potential role of SLFN5 in the anti-tumor effects of IFN-α in humans. Unlike the observations in malignant melanoma and RCC, SLFN5 inhibits the anti-tumor effect of interferon in glioma multiforme cells by binding and co-repressing STAT1 stimulation. Knockdown of SLFN5 enhances cellular sensitivity to IFN-induced antiproliferative responses in glioma stem-like cancer cells, making SLFN5 a negative regulator of the IFN-response in glioma cancer cells [52]. IFN also upregulates the expression of SLFN11, SLFN12, SLFN13, and SLFN14 in various benign and malignant neural cells, but whether these upregulated SLFNs contribute to the activity of the interferon has not yet been studied [52].

An extensive study is required to dissect the role of each Schlafen in response to interferons therapy in each cancer subtype, and to identify the magnitude of interferon anti-tumor response in high Schlafen expressing vs. low expressing tumors, which may predict the patients who could benefit from interferon therapy.

## 19. Role of Human Schlafens in Cancer Chemosensitivity

### 19.1. Role of Schlafen 11 in Chemosensitivity

In vitro observations have implicated SLFN11 as a potential marker of cancer cell chemosensitivity to agents as diverse as PARPi [69,101,102,103,104,105,106], platinum-based drugs [21,65,69,94,107], topoisomerase inhibitors [18,108,109,110,111,112], and antibody-drug conjugates [112] in a variety of tumor types including breast [67,94,101], lung [21,67,101,103,104,107], ovarian [21,67], prostate [69,101], colon [101,108], Ewing sarcoma [101,109], gastric carcinoma [65], and leukemias [108,111]. Mouse studies showed that high SLFN11 expression increases the synergistic effect of talazoparib-temozolomide in small cell lung carcinoma xenografts [101]. Moreover, in xenografts derived from triple negative breast cancer patients, SLFN11 has been identified in the top upregulated genes in irinotecan responders compared to the resistant xenografts [110]. High SLFN11 expression also predicts sensitivity to doxorubicin in these xenografts [110]. High SLFN11 expression in prostate cancer patient-derived xenografts also increases their sensitivity to LMP400 [111]. Indeed, in humans, epidemiologic data suggest that SLFN11 expression is higher in patients with ovarian, lung, or breast cancer who respond to chemotherapy [67] and in patients with gastric carcinoma who survive longer [65].

### 19.2. Role of Schlafen 12 in Chemosensitivity

Drug resourcing has identified SLFN12 as a target of 6-(4-(diethylamino)-3-nitrophenyl)-5-methyl-4,5- dihydropyridazin-3(2H)-1 (DNMDP). DNMDP promotes phosphodiesterase 3A (PDE3A) physical interaction with SLFN12 and induces apoptosis in 766 cancer cell lines [113,114], and depleting SLFN12 decreases the DNMDP sensitivity [114]. DNMDP-derived molecule (R)-30/BRD9500 induces PDE3A/SLFN12 interaction in cervical adenocarcinoma HeLa cells to promote apoptosis [115]. Also, this molecule has shown activity with oral dosing in melanoma xenografts [115]. High SLFN12 expressing cancer cells are more sensitive to PDE3 inhibitors (zardaverine and quazinone) than low SLFN12 expresser cells [116]. Additionally, the sensitivity to zardaverine and quazinone (PDE3 inhibitors) in primary cultures of patient-derived ovarian cancer correlates with both SLFN12 and PDE3A expression [116]. Interestingly, SLFN12 induces apoptosis in Hela cells (cervical adenocarcinoma) in response to 17-ß-estradiol (E2) treatment [83]. Upon 17-ß-estradiol binding to phosphodiesterase 3A (PDE3A), PDE3A in turn recruits, binds, and stabilizes SLFN12. In turn, SLFN12 inhibits the translation of antiapoptotic proteins, and consequently activates the mitochondrial pathway of apoptosis [83]. Similar to 17-ß-estradiol, nauclefine (an indole alkaloid natural product) also induces PDE3A-SLFN12-dependent apoptosis in HeLa cells [117].

SLFN12 mutations can reduce DNMDP sensitivity even in the presence of high PDE3A expression, as noted in CAL51 cells, which harbor heterozygous SLFN12 nonsense gene mutation at amino acid 196 [118]. Moreover, a SLFN12 (I105N) mutation prevents SLFN12-PDE3A interaction and abolishes the cell death effect of nauclefine in Hela cells [117].

## 20. Conclusions

Although it has been more than twenty years since Schlafens were discovered, they remain intriguing proteins and are still enigmas. The exploration of the role of Schlafens in cancers is still in its early stages and evidence predicts a considerable role for Schlafens in tumorigenesis, as biomarkers and predictors for chemotherapy, and as possible targets for drugs. 

The role of Schlafens is not universal, and each Schlafen has a distinct specific mechanism through which it affects cancer cell biology. Interestingly, this mechanism is not alike in all cancer types/subtypes. Instead, Schlafens demonstrate different effects in different subtypes of tumors of the same organ.

The complex signaling networks through which Schlafens are reported to act in cancer urges extensive future characterization and exploration of each Schlafen in the different cancer subtypes, which will eventually push us closer to better understand cancers in humans.

## Figures and Tables

**Table 1:** Summary of the roles of human Schlafens in different cancer types. Each Schlafen expression level and the correlation with survival were identified. The non-cited data are derived from our analysis of publicly available tools (https://www.proteinatlas.org, accessed on 10 July 2021) and (https://kmplot.com/analysis/, accessed on 10 July 2021).

Cancer	Schlafen	Expression Level	Survival Correlation
Malignant Melanoma	SLFN5	Downregulated [62].	Positive [62].
	SLFN11	No significant change.	Negative.
	SLFN12	No significant change.	Negative.
	SLFN14	No significant change.	Positive.
Renal Carcinoma	SLFN5	Downregulated.	Positive [63].
	SLFN11	Downregulated [64].	Positive [64].
	SLFN12	Downregulated.	Negative.
	SLFN14	Downregulated.	Negative.
Gastric Carcinoma	SLFN5	Upregulated [16].	Negative [16].
	SLFN11	Downregulated [65].	Positive [65].
	SLFN12	Upregulated.	Not prognostic.
	SLFN14	Downregulated.	Not prognostic.
Colorectal Carcinoma	SLFN5	No significant change.	Negative.
	SLFN11	Downregulated [22,64].	Positive [22].
	SLFN12	Downregulated.	Positive.
	SLFN14	Downregulated.	Not prognostic.
Lung Carcinoma	SLFN5	Downregulated [64,66].	Positive [66].
	SLFN11	Downregulated [22].	Positive [67].
	SLFN12	Downregulated [15].	Positive [15].
	SLFN14	Downregulated.	Positive in lung adenocarcinoma.
Prostate Carcinoma	SLFN5	Upregulated [68].	Negative [68].
	SLFN11	Upregulated in metastatic prostate cancer [69].	No correlation to O.S., but positive correlation to rPFS [69].
	SLFN12	Downregulated.	Not prognostic.
	SLFN14	Downregulated.	Not prognostic.
Liver Cancer	SLFN5	Downregulated [70].	Positive [70].
	SLFN11	Downregulated [71].	Positive [71].
	SLFN12	Not Significant.	Negative.
	SLFN14	Downregulated.	Not prognostic.
Esophageal Cancer	SLFN5	Upregulated.	No data available.
	SLFN11	Downregulated with age [72].	Positive [72].
	SLFN12	Upregulated	No data available.
	SLFN14	No significant change.	No data available.
Breast Cancer	SLFN5	Downregulated [73,74].	Positive [73,74].
	SLFN11	Downregulated.	Positive/negative after hormone therapy [17].
	SLFN12	Downregulated [14].	Positive in triple negative breast cancer [14].
	SLFN14	Downregulated.	Positive.
CNS Tumors	SLFN5	Upregulated [52].	Negative [52].
	SLFN11	Upregulated [52].	Negative [52].
	SLFN12	Upregulated [52].	Negative [52].
	SLFN13	Upregulated [52].	Negative [52].
Leukemia	SLFN5	No significant change.	No data available.
	SLFN11	Upregulated.	No data available.
	SLFN12	Upregulated.	No data available.
	SLFN14	Upregulated.	No data available.
